# Machine learning determination of motivators of terminal extubation during the transition to end-of-life care in intensive care unit

**DOI:** 10.1038/s41598-023-29042-9

**Published:** 2023-02-14

**Authors:** Petr Waldauf, Nathan Scales, Jason Shahin, Matous Schmidt, Amanda van Beinum, Laura Hornby, Sam D. Shemie, Melania Hogue, Tineke J. Wind, Walther van Mook, Sonny Dhanani, Frantisek Duska

**Affiliations:** 1 Department of Anaesthesia and Intensive Care Medicine, Third Faculty of Medicine, Charles University and University Hospital Královské Vinohrady, Prague, Czech Republic; 2 Ottawa Hospital Research Institute, Ottawa, ON Canada; 3 Faculty of Medicine, Division of Critical Care, Respiratory Epidemiology and Clinical Research Unit, McGill University Health Centre, Montreal, QC Canada; 4 Children’s Hospital of Eastern Ontario Research Institute, Ottawa, ON Canada; 5 Pediatric Critical Care, Montreal Children’s Hospital, Montreal, QC Canada; 6 Canadian Donation and Transplant Research Program, Edmonton, Canada; 7 System Development, Canadian Blood Services, Ottawa, ON Canada; 8 Maastricht University Medical Centre and Heart and Vascular Centre, Maastricht, The Netherlands; 9 Department of Intensive Care Medicine, Maastricht University Medical Centre, and School of Health, Maastricht, The Netherlands

**Keywords:** Medical research, Outcomes research

## Abstract

Procedural aspects of compassionate care such as the terminal extubation are understudied. We used machine learning methods to determine factors associated with the decision to extubate the critically ill patient at the end of life, and whether the terminal extubation shortens the dying process. We performed a secondary data analysis of a large, prospective, multicentre, cohort study, death prediction and physiology after removal of therapy (DePPaRT), which collected baseline data as well as ECG, pulse oximeter and arterial waveforms from WLST until 30 min after death. We analysed a priori defined factors associated with the decision to perform terminal extubation in WLST using the random forest method and logistic regression. Cox regression was used to analyse the effect of terminal extubation on time from WLST to death. A total of 616 patients were included into the analysis, out of which 396 (64.3%) were terminally extubated. The study centre, low or no vasopressor support, and good respiratory function were factors significantly associated with the decision to extubate. Unadjusted time to death did not differ between patients with and without extubation (median survival time extubated vs. not extubated: 60 [95% CI: 46; 76] vs. 58 [95% CI: 45; 75] min). In contrast, after adjustment for confounders, time to death of extubated patients was significantly shorter (49 [95% CI: 40; 62] vs. 85 [95% CI: 61; 115] min). The decision to terminally extubate is associated with specific centres and less respiratory and/or vasopressor support. In this context, terminal extubation was associated with a shorter time to death.

## Introduction

Worldwide, 15–30% of patients admitted to intensive care (ICU) die in hospital^1^. A large proportion of these deaths are preceded by withholding or withdrawing life-sustaining treatment (WLST) that is no longer considered to be of benefit for the dying patients. Globally, withholding treatment is the most common form of limiting care, albeit with a large regional variability^2^. However, the representation of withdrawal has recently increased and in some regions it is already the dominant form of limitation of LST^3^. In addition to withdrawal of circulatory support, all non-comfort medications, nutrition, dialysis and mechanical ventilation (MV) are usually also withdrawn. Procedural aspects of withdrawal of MV are understudied, but its most common forms are either terminal weaning (a gradual reduction of ventilatory support, such as reducing the inspiratory FiO_2_, positive end-expiratory pressure, minute ventilation or switching the patient to spontaneous ventilation, whilst the endotracheal cannula is left in place) or as immediate removal of the endotracheal tube (further referred to as terminal extubation, TE)^4^.

TE in patients nearing the end of life in intensive care can prevent gagging and facial distortion of patients, thereby increasing the perceived comfort and dignity of dying. On the other hand, it may lead to grunting and/or gasping due to the loss of airways^5^, and there are concerns that it may hasten death. Current European guidelines recommend an individualized approach to ensure patient comfort^6^ despite the fact that the impact of extubation on patients, their families and the psychological well-being of healthcare providers may be difficult to predict in each individual case^5^. In practice, the decision on technical aspects of terminal care is often made based on local practices, following consensus among staff and relatives. To what extent these measures are tailored to patients’ needs may also significantly differ among centres. It is unknown whether specific patient characteristics may influence (consciously or subconsciously) healthcare providers to decide whether to perform TE or not. These characteristics are likely to have a complex non-linear relationship that can be explored using machine learning methods.

We sought to determine: (1) factors associated with the decision to perform TE as part of withdrawal of life-sustaining therapy (WLST); and (2) whether this action influences the time to death after these factors have been taken into consideration.

## Methods

The reporting of this study is in accordance with the Strengthening the Reporting of Observational studies in Epidemiology (STROBE) statement^7^.

### Study design

We performed a post hoc secondary analysis of data collected as part of a large, multi-national, prospective, observational study on the process of WLST in dying patients (the Death Prediction and Physiology after Removal of Therapy, or DePPaRT study), which collected baseline data and ECG, pulse oximeter and arterial waveforms from WLST until 30 min after determination of death. Patients in participating intensive care units in Canada, the Czech Republic, and the Netherlands were enrolled between May 2014 and December 2018. The DePPaRT study protocol, including secondary data analyses, were approved by the relevant institutional review board or ethics committee at each site (see Supplementary Table S8), and all patients’ surrogate decision makers provided written prospective informed consent for participation in the study. The protocol is compliant with the Declaration of Helsinki and data storage and analyses were performed in accordance with national legislation and General Data Protection Regulation of the European Union.

### Patients

A subgroup of DePPaRT study patients (age ≥ 18 years who died after WLST in an intensive care unit) who had their lower airway secured with an orotracheal or tracheostomy cannula were included into the analysis. As per the DePPaRT study protocol, patients were excluded if they had a neurological determination of death, a functional cardiac pacemaker, or no arterial catheter at the time of WLST. Patients who died in the ICU due to unsuccessful cardiopulmonary resuscitation were also excluded from the study.

### Data collection

**Figure 1:**
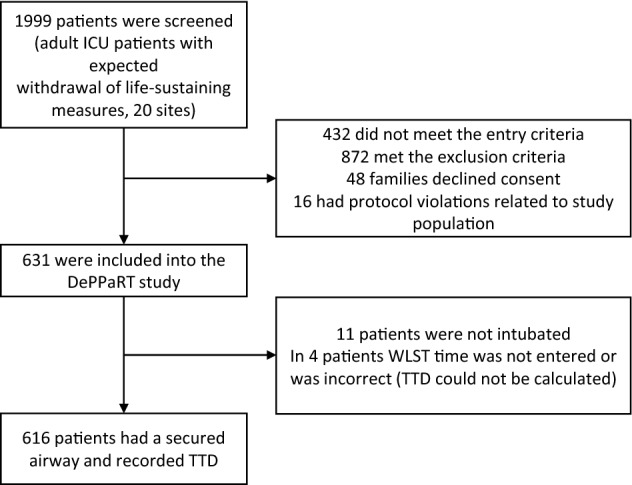
Flowchart of the study enrolment. Note: TTD = time to death; WLST = withdrawal of life-sustaining treatments.

For this secondary analysis a subset of available parameters (features) was selected for the machine learning model based on the literature study and consensus of 3 experts (PW, MS, FD). We also, a priori, decided to exclude parameters with more than 20% missing values (Fig. 1).

The following data were included in the data set:

The dependent variable was TE (yes vs. no). Independent variables used for modelling (N = 28) included:

Patients’ characteristics at baseline: study centre (binarized at 50% of terminal extubation frequency, group1 <  = 50%, group2 > 50%), age, sex, body mass index (BMI), acute physiology and chronic health evaluation II score at admission (APACHE)^8^, chronic pre-existing medical condition (PreCond, yes/no), cardiac arrest with resuscitation before study inclusion (CPR, yes/no), admission diagnosis (3 most common categories one-hot encoded into dummy features: neurologic disorder (ADM_neuro), respiratory failure (ADM_resp), sepsis (ADM_sepsis); yes/no).

Patients’ characteristics at WLST: Glasgow coma scale (GCS), pupillary reflex (PUP, present vs. absent), cough (present vs. absent), mechanical ventilation mode (MV mode, controlled vs. supported), respiratory rate (RR, bpm), inspiratory fraction of oxygen (FiO_2_%), positive end expiratory pressure (PEEP, cmH_2_O), peak inspiratory pressure (PIP, cmH_2_O), intubation route (route, orotracheal intubation vs. tracheostomy), mean arterial pressure (MAP, mmHg), hear rate (HR, bmp), lactate (mmol L^−1^), arterial pH, arterial partial pressure of oxygen (PaO_2_, mmHg), arterial partial pressure of carbon dioxide (PaCO_2_, mmHg), total ranked circulatory drugs dose (circ_total_ranked), total ranked sedation drugs dose (sedation_total_ranked) and total ranked analgesics drugs dose at WLST (analgetics_total_ranked). Attempted donation after circulatory death was collected (DCD, yes/no).

### Statistical analysis

All analyses and data processing were performed in R v 4.2.1^9^ and RStudio v 2022.02.3^10^. Exploratory data analysis was calculated for all parameters. Univariate analysis was done using Wilcoxon rank sum test for continues and Pearson’s Chi-squared test for categorical features.

### Data pre-processing

Features were kept in their original form to simplify the interpretation of the eventual nonlinear relationship between a feature and the probability of TE.

Normalization was performed only for drugs so that we could combine doses of different drugs in the same drug group (circulatory, sedation and analgesics drugs). To normalize doses, we used a rank doses method similar to that described by Trace et al^11^. Patients were ranked for each drug from lowest to highest dose administered in the hour prior to withdrawal. The rank was then divided by the total number of patients who received that drug. This gave the relative rank dose for each drug. The relative rank doses of the drugs in each drug group were then summed. This gave the total rank dose for each drug group. The total ranked dose of circulatory drugs was calculated as the sum of ranked doses of norepinephrine, epinephrine, vasopressin, and phenylephrine. Similarly, we calculated the total ranked sedation drug dose (midazolam and propofol) and the total ranked analgesics drugs dose (morphine, fentanyl, and hydromorphone).

### Data imputation

Missing data (both continuous and categorical) were imputed using a random forest model, package missForest v 1.4^12^.

### Machine learning (ML) analysis

Mlr3 toolbox was used for ML modelling (mlr3verse package v. 0.2.5^13^). Two classification ML models were used: logistic regression (LR) and random forest (RF). LR was chosen as a "glass box" that is well interpretable and known to the general medical community. RF was chosen as a robust "black box", capable of modelling even complex non-linear relationships and interactions between features while not requiring normalization or scaling of features.

For RF with number of trees, maximal tree depth and minimal node size hyperparameter tuning, we used package ranger v. 0.14.1^14^. LR was performed with feature selection (Lasso regularization (s to z), package glmnet 4.1-4^15^). For hyperparameter tuning we used 5-fols cross-validation with classification error (CE) as performance measure and for the model performance evaluation (CE, ROC AUC, Brier score^16^) 5-fold cross-validation repeated 5 times. The best performing model (for both RF and LR) was used to create the final model on the full dataset.

Feature importance, measured as the factor by which the model's prediction error increases when the feature is shuffled, was calculated^17^. Accumulated local effects (ALE) plots^18^, describing how features influence the prediction of a machine learning model (terminal extubation) on average and independently on other features, were created for individual features of random forest model using iml package 0.10.1^19^. Finally overall interaction strength for each feature with all other features was calculated.

### Survival analysis

Survival analysis was performed using univariate and multivariate Cox regression with feature selection using Lasso regularization (s to z), (package glmnet 4.1-4^15^) with all features mentioned above. Adjusted survival curves and adjusted median with 95% confidence interval were created using package adjustedCurves 0.9.0^20^.

## Results

Six hundred and sixteen patients from 20 centres in Canada (N = 355, 57.6%), the Czech Republic (N = 219, 35.6%) and the Netherlands (N = 42, 6.8%) were included in the analysis (see flowchart Fig. 1). The most common admission diagnosis was neurologic disorder (48.5%), respiratory failure (15.1%) and sepsis (14.3%). Further patient characteristics along with univariate comparisons of individual features between extubated and non-extubated patients are shown in Table 1 and Supplementary Tables S1–S4. Three hundred and ninety-six (64.3%) patients were terminally extubated. Eighty-seven patients (14.1%) underwent an attempt to organ donation after circulatory determined death (also known as donation after cardiac death, DCD) and sixty (9.7% of total sample, 69% of DCD attempted) proceeded to DCD. The median time from initiation of WLST to death was 1 h (IQR: 0.3–4.7 h). Correlation between features can be seen in Supplementary Fig. S3. Multicollinearity was tested by variance inflation factor (VIF) and was low (below 3) for all features.

**Table 1:** Characteristics of enrolled patients (N = 616) (imputed data), stratified by terminal extubation.

Feature	All patients (N = 616)	Without terminal extubation N = 220 (35.7%)	Terminal extubation N = 396 (64.3%)	*p* value
**Characteristics of enrolled patients at baseline**				
**Country**				
Canada	355 (57.6%)	66 (30.0%)	289 (73.0%)	< 0.001**
Czech Republic	219 (35.6%)	153 (69.5%)	66 (16.7%)	
Netherlands	42 (6.8%)	1 (0.5%)	41 (10.4%)	
**Centre**				
Group 1	271 (44.0%)	187 (85.0%)	84 (21.2%)	< 0.001**
Group 2	345 (56.0%)	33 (15.0%)	312 (78.8%)	
Age	65 (55.6, 74.6)	66 (57, 75)	64 (55, 74)	0.14*
**Sex**				
Females	233 (37.8%)	94 (42.7%)	139 (35.1%)	0.061**
Males	383 (62.2%)	126 (57.3%)	257 (64.9%)	
Chronic pre-existing medical condition	507 (82.3%)	197 (89.5%)	310 (78.3%)	< 0.001**
Cardiac arrest with resuscitation before study inclusion	84 (13.6%)	40.0 (18.2%)	44.0 (11.1%)	0.014**
**Admission diagnosis**				
Neurologic disorder	303 (49.2%)	66.0 (30.0%)	237.0 (59.6%)	< 0.001**
Respiratory failure	94 (15.3%)	45.0 (20.5%)	49.0 (12.4%)	0.008**
Sepsis	91 (14.8%)	51.0 (23.2%)	40.0 (10.1%)	< 0.001**
Other	128 (20.8%)	58.0 (26.4%)	71.0 (17.9%)	0.014**
Active malignancy^†^	96.0 (15.6%)	50.0 (22.7%)	46.0 (11.6%)	< 0.001**
Metastatic malignancy^†^	30.0 (4.9%)	14.0 (6.4%)	16.0 (4.0%)	0.2**
BMI	27 (24, 31)	28 (23, 31)	27 (24, 30)	0.9*
APACHE II score (1st 24 h at ICU)	27 (22, 32)	29 (22, 35)	27 (22, 31)	0.043*
**Characteristics of enrolled patients at WLST**				
GCS	3 (3, 5)	3 (3, 3)	3 (3, 6)	< 0.001*
**Pupillary reflex**				
Present	428 (69.5%)	169 (76.8%)	259 (65.4%)	0.003**
Absent	188 (30.5%)	51 (23.2%)	137 (34.6%)	
Cough reflex				
Present	449 (72.9%)	161 (73.2%)	288 (72.7%)	> 0.9**
Absent	167 (27.1%)	59 (26.8%)	108 (27.3%)	
**Ventilation mode**				
Support	175 (28.4%)	41 (18.6%)	134 (33.8%)	< 0.001**
Control	441 (71.6%)	179 (81.4%)	262 (66.2%)	
Respiratory rate [bpm]	20 (16, 25)	20 (18, 26)	20 (15, 24)	0.009*
FiO_2_ [%]	40 (30, 60)	50 (35, 71)	40 (30, 50)	< 0.00*
PEEP [cmH_2_O]	8 (6, 10)	8 (6, 10)	8 (5, 10)	0.6*
Peak inspiratory pressure [cmH_2_O]	22 (18, 27)	24 (18, 30)	22 (18, 26)	< 0.001*
**Route**				
Endotracheal tube	597 (96.9%)	208 (94.5%)	389 (98.2%)	0.011*
Tracheostoma	19 (3.1%)	12 (5.5%)	7 (1.8%)	
Mean arterial pressure [mmHg]	73 (62, 90)	65 (53, 76)	79 (68, 96)	< 0.001*
Heart rate [bpm]	91 (75, 108)	98 (80, 114)	88 (74, 105)	< 0.001*
Lactate [mmol L^−1^]	1.8 (1, 3.8)	3.0 (1.3, 10.2)	1.5 (1.0, 2.3)	< 0.001*
pH (arterial)	7.39 (7.28;7.45)	7.31 (7.20, 7.40)	7.42 (7.36, 7.46)	< 0.001*
pO_2_ [mmHg] (arterial)	95 (76, 118)	90 (71, 107)	100 (80, 128)	< 0.001*
pCO_2_ [mmHg] (arterial)	39 (34, 45)	41 (34, 48)	38 (34, 43)	0.008*
Total ranked dose of circulatory drugs	0.05 (0, 0.61)	0.59 (0.06, 0.86)	0 (0, 0.25)	< 0.001*
Patients with circulatory drugs^†^	325.0 (52.8%)	171.0 (77.7%)	154.0 (38.9%)	< 0.001**
Total ranked dose of circulatory drugs (only patients with circulatory drugs)^†^	0.59 (0.26, 0.85)	0.71 (0.45, 0.92)	0.36 (0.18, 0.74)	< 0.001*
Total ranked dose of sedatives	0 (0, 0.37)	0 (0, 0.31)	0 (0, 0.42)	0.2*
Patients with sedatives^†^	270.0 (43.8%)	88.0 (40.0%)	182.0 (46.0%)	0.2**
Total ranked dose of sedatives (only patients with sedatives)^†^	0.47 (0.22, 0.81)	0.44 (0.23, 0.79)	0.47 (0.20, 0.81)	> 0.9
Total ranked dose of opioids	0.04 (0, 0.49)	0 (0, 0.45)	0.10 (0, 0.51)	0.051*
Patients with opioids^†^	313.0 (50.8%)	100.0 (45.5%)	213.0 (53.8%)	0.047**
Total ranked dose of opioids (only patients with opioids)^†^	0.48 (0.26, 0.80)	0.48 (0.25, 0.80)	0.49 (0.26, 0.79)	0.7*
Eligible for donation after circulatory death^†^	307.0 (49.8%)	68.0 (30.9%)	239.0 (60.4%)	< 0.001**
Attempted donation after circulatory death	87 (14.1%)	4 (1.8%)	83 (21.0%)	< 0.001*
Successful donation after circulatory death	60 (9.7%)	4 (1.8%)	56 (14.1%)	< 0.001*

Continues features: median (IQR), *Wilcoxon rank sum test, categorical features: n (%); **Pearson’s Chi-squared test; ^†^Parameters that are not part of the models.

The performance of the RF and LR model with the five-fold cross-validation on the test set was similar, with an average ROC AUC of 0.91 and 0.90, classification error 16.6 and 16.4% (see Supplementary Fig. S4 and Supplementary Table S6).

**Figure 2:**
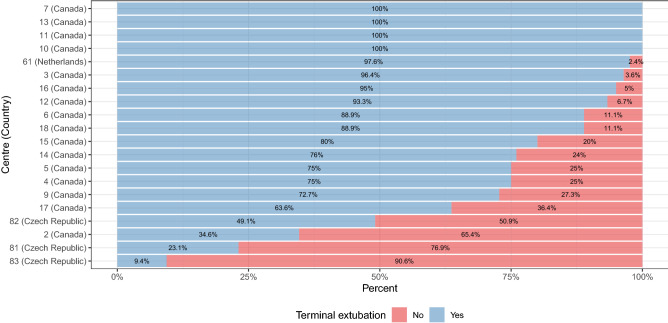
Frequency of terminal extubation across centres. Y-axis: study centre ID and country. Note: Group 1 includes all centres in the Czech Republic and one Canadian centre. Group 2 includes all remaining centres in Canada and the centre in the Netherlands.

By far the most important feature in both models was the study centre (binarized by frequency of terminal extubation). Frequency of TE across centres is shown in Fig. 2. The proportion of patients with TE was 97.6% in the Netherlands, 81.4% in Canada and 30.1% in the Czech Republic. Four Canadian centres extubated all patients enrolled at their site. There was no centre in the study that did not at all perform TE.

**Figure 3:**
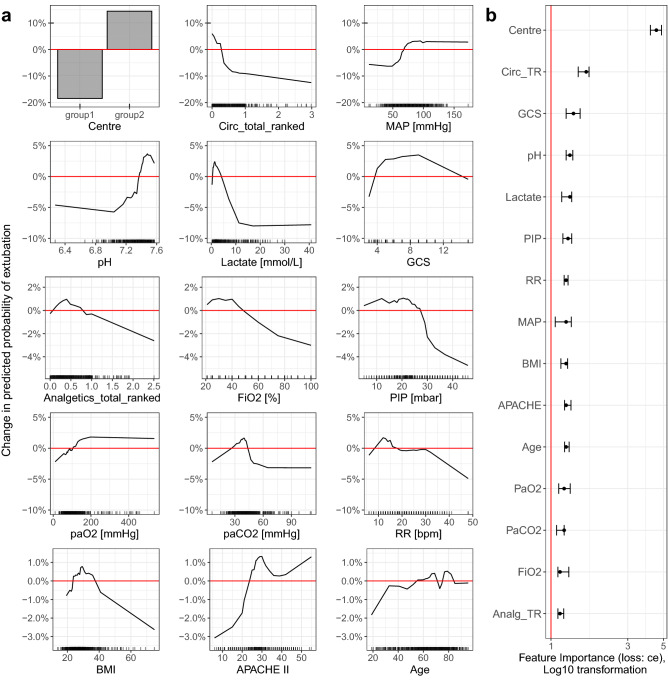
Visual interpretation of the random forest model for 15 most important features: (**a**) Accumulated local effects plots describing how features influence the prediction of a RF model (terminal extubation) on average (red horizontal line). Y-axis: % change in probability of extubation. The validity of the curves is limited in areas with few data—see the rug plot on the x-axis. The corresponding partial dependence plots (PDP) curves are shown in Fig. S5. (**b**) Permutation feature importance measured as the factor by which the model’s classification error (CE) increases when the feature is shuffled in the test data (permuted CE/original CE [4.7%]).

Visual interpretation of RF model (15 of the 28 most important features) can be seen in Fig. 3 and feature importance of all features in Supplementary Table S7.

Other important patient characteristics associated with likelihood of TE, include the “circulation status” and the “respiratory status” features at the time of WLST. Patients on higher vasopressor support, lower mean arterial pressure, higher lactate, and lower pH are less likely to be terminally extubated. Similarly, patients with higher peak inspiratory pressure, higher FiO_2_, lower paO_2_, higher paCO_2_ and higher respiratory rate (i.e., in respiratory failure) are also less likely to be extubated. The most important parameters in this group are FiO_2_ and peak inspiratory pressure. Lastly, patients on a higher dose of opioids or with GCS of 3 are less likely to be extubated.

The output of the LR with Lasso feature selection is shown in Supplementary Table S5. LR captures the same patterns as RF with the centre being the strongest feature, and features describing the severity of circulatory and respiratory failure as negative prognostic markers of TE. In addition, LR selected a route where tracheostomy patients have a lower chance of TE.

**Figure 4:**
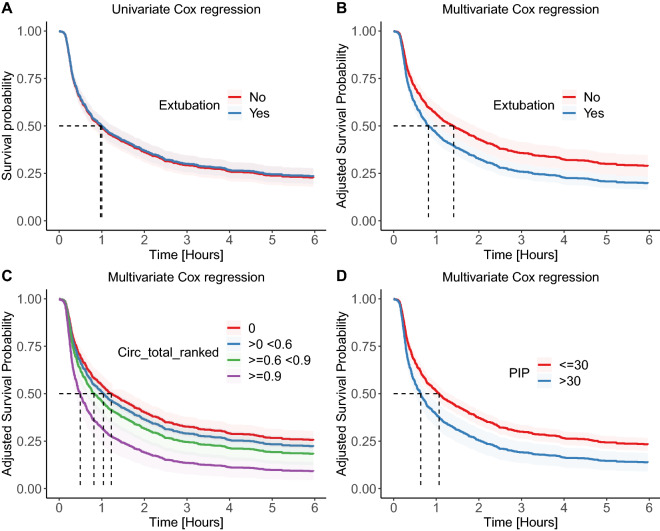
Plot of survival after WLST derived from the Cox regression model. (**a**) Univariate Cox regression comparing patients with and without terminal extubation. (**b**) Adjusted Cox regression comparing patients with and without terminal extubation. (**c**) Adjusted Cox regression comparing patients with different total circulatory drugs doses. (**d**) Adjusted Cox regression comparing patients with peak inspiratory pressure above and below 30 cmH_2_O. Note: Circ_total_ranked = total ranked circulatory drugs dose, PIP = peak inspiratory pressure.

Time to death after WLST did not differ between patients with and without TE (univariate Cox regression: HR 0.98 (95% CI: 0.81; 1.18), *p* = 0.83, median survival time extubated vs. not extubated: 60 [95% CI: 46; 76] vs. 58 [95% CI: 45; 75] min). After adjustment for confounders, time to death was significantly shorter in patients with TE (multivariate Cox regression: adj. HR 1.46 [95% CI 1.11; 1.92], *p* = 0.007, median survival time extubated vs. not extubated: 49 [95% CI: 40; 62] vs. 85 [95% CI: 61; 115] min), see Table 2 and Fig. 4.

**Table 2:** Multivariate Cox regression with feature selection (Lasso regularization (s to z)).

Feature	Adjusted hazard ratio (95% confidence interval)	*p* value
Extubation (yes vs. no)	1.46 (1.11; 1,92)	0.007
**Ranked total circulatory drugs dose at WLST reference = 0**		
> 0 and < 0.6	1.14 (0.90; 1.45)	0.296
≥ 0.6 and < 0.9	1.35 (0.98; 1.85)	0.068
≥ 0.9	2.19 (1.50; 3.18)	< 0.001
Centre (Group 2 vs. Group 1)	1.61 (1.24; 2.09)	< 0.001
Attempted donation after circulatory death (yes vs. no)	1.63 (1.22; 2.19)	< 0.001
Glasgow Coma Scale (cont.)	0.91 (0.86; 0.96)	< 0.001
Pupillary reflex (absent vs. present)	1.34 (1.08; 1.66)	0.008
Cough reflex (absent vs. present)	1.23 (0.989; 1.53)	0.063
Peak inspiratory pressure [cmH_2_O] (> 30 vs. ≤ 30)	1.54 (1.17; 2.03)	0.002
Mechanical ventilation mode (controlled vs. supported)	1.37 (1.08; 1.73)	0.01
FiO_2_ (cont.)	1.005 (1.0; 1.009)	0.034
Mean arterial pressure [mmHg] (cont.)	0.986 (0.981; 0.991)	< 0.001
Heart rate [bpm] (cont.)	1.007 (1.003; 1.011)	< 0.001
pH (cont.)	0.31 (0.15; 0.36)	< 0.001
Total ranked dose of opioids (cont.)	1.41 (1.12; 1.76)	0.003
BMI (cont.)	1.016 (1.003; 1.029)	0.019
APACHE II score (cont.)	1.011 (0.999; 1.023)	0.076

## Discussion

In this study we sought to determine which factors influence health care providers' decision to perform TE at the end of life and whether the TE influences time to death. Our analyses demonstrate that the probability of TE was influenced more by the study centre than by patients’ characteristics, suggesting that local protocols or habits may dominate over individualisation of care according to individual patients’ needs. This is consistent with the findings of the large epidemiological studies Ethicus-1^21^ and Ethicus-2^2^, which described significant regional differences in the way life-sustaining treatment is limited in the ICU. Yet, some patient-related factors were indeed associated with the probability of TE. Most importantly, patients without circulatory or respiratory failure are more likely to be extubated.

The reason behind this pattern might be the belief that unstable patients are expected to die shortly after the withdrawal of vasopressors and/or ventilatory support, whereas in more stable patients, healthcare providers may be concerned about potential suffering during protracted dying, perhaps making them more likely to perform TE. The survival curves of patients with and without TE follow almost identical trajectories. Without knowing the factors that had influenced the decision to perform TE, this could be interpreted as that TE does not influence time to death, which was also the conclusion of previous observational studies by Suntharalingam et al.^22^ or Wind et al.^23^. Of note, the effect of TE on time to death becomes apparent and significant after adjustment for the factors that are associated with the decision to perform TE. Even though the death-hastening effect of TE is still smaller than the effect of withdrawal of high doses of vasopressors (Fig. 4 and Supplementary Fig. S8), we believe it is of clinical importance. Nonetheless, the finding that patients on low doses of opioids are more likely to be extubated may reflect healthcare providers’ intention to sustain or restore spontaneous breathing efforts before the intended TE, which argues against conscious or subconscious intention to shorten the process of dying. It also may reflect the fact that healthcare providers believe the removal of endotracheal tube will relieve patients’ suffering that would otherwise require the administration of opioids.

Another interesting finding is that although over 95% of patients with attempted DCD were terminally extubated, this parameter was eliminated from both multivariate analyses (both RF and regularized LR). A possible explanation is that the patients in whom DCD was attempted had predominantly neurological injuries and were more likely to have normal gas exchange and circulation, i.e., features typical for patients that are terminally extubated. Therefore, at least from the point of view of TE, healthcare providers did not treat the DCD cohort differently. On the other hand, attempted DCD remains a significant parameter in multivariate Cox regression and these patients die faster.

Interpretation of the individual influence of each feature on TE can be aided by principal component analysis (PCA), which supports the above explanations and can be found in the Supplementary appendix (see Supplementary Fig. S9).

Investigation of technical aspects of WLST is very difficult, and to the best of our knowledge, there are no randomised controlled trials in the field. Answering important questions such as what the effects of and for the TE motivators are, relies on observational data. Our study represents a multi-centre, multi-national, observational trial with the largest sample size so far published. Such a sample allowed us to use state-of-the-art machine learning techniques to explore the complex and nonlinear relationships between variables associated with TE. Of note, the performance of RF and regularized LR was very similar. The high performance of LR is due to the low interaction rate of individual features (Supplementary Fig. S6). We have chosen RF as the main model because it allows an intuitive visualization of very complex relationships between TE and features using ALE (Fig. 3) and partial dependence plots (PDPs) (Supplementary Fig. S5). In addition, RF can automatically detect interactions between features. ALE plots are an unbiased alternative to the PDPs^24^, which means they still work even when features are correlated^25^. On the other hand, ALE plots can produce misleading interpretations when features strongly interact^26^, which is not the case here. The validity of both plots is limited in areas with few data. All multivariate models used require a dataset with no missing data. Otherwise, the model eliminates subjects with missing data which may cause selection bias and reduce the power of the analysis. For this reason, missing data were imputed under the assumptions that they are missing at random. Many machine learning algorithms require transformation of continuous parameters in the form of normalization or standardization. Our goal was not to create the best possible predictive model, but to explore the potential relationship of each parameter under study to TE while maximizing interpretability, which is limited if the parameters are transformed. For this reason, we limited ourselves to normalizing the drugs so that their doses could be summarized across drug groups. For the same reason, we used a random forest algorithm that is robust to untransformed data. An objection could be that we only used internal and not external validation. This was because of the limited number of patients and the primarily exploratory not predictive purpose of our work. Thus, the performance of the models as presented in Supplementary Table S8 and Supplementary Fig. S4 will be overestimated compared to how it would look if performed on an external data set on which the models were not trained.

From the clinical perspective, it is important that TE is likely to have the potential to hasten death in stable ICU patients at the end of life, but to a smaller extent than withdrawing the vasopressors in unstable patients. This information may help healthcare providers to tailor the technical aspects of compassionate care to patients’ and families’ wishes and values, including the decision to proceed with DCD.

The primary limitation of our study is its non-randomised nature, which means that despite the sophisticated methodology, the discovered relations will always remain associative, without conclusive evidence of the causality. The cohort of extubated patients also included patients with tracheostomy who were decannulated (n = 19, 3.1%), but these numbers are too small to allow generalisability of our results to all patients with cuffed airways. In addition, this secondary data analysis was not specified a priori and therefore the spectrum of the analysed features is limited and may not include all motivators to TE. Lastly, we made assumptions about healthcare providers’ motivators to perform TE, based on quantitative data, without directly interviewing them. Future research should use qualitative methodology to gain a deeper insight into the conscious motivators of healthcare providers to perform TE and possibly explore other ML-based methods^27^.

In conclusion, the decision to terminally extubate is associated with specific centres and less respiratory and/or vasopressor support. In this context, terminal extubation was associated with a shorter time to death.

## Supplementary Information

Supplementary Information.

## Data Availability

De-identified record level data will be provided by the authors upon reasonable request. To request data from this study, please contact Dr. Petr Waldauf by email: petr.waldauf@lf3.cuni.cz.

## Abbreviations

CE: Classification error
DCD: Donation after circulatory death
DePPaRT: Death prediction and physiology after removal of therapy
ICU: Intensive care unit
LR: Logistic regression
LST: Life-sustaining treatment
ML: Machine learning
RF: Random forest
TE: Terminal extubation
WLST: Withdrawal of life-sustaining therapy
