# Supplementary material for: Machine learning determination of motivators of terminal extubation during the transition to end-of-life care in intensive care unit
Source: Sci Rep. 2023 Feb 14;13:2632. doi: 10.1038/s41598-023-29042-9 (PMC9929077; doi:10.1038/s41598-023-29042-9)
Supplement: Supplementary file 1 — Supplementary Information. [file 41598_2023_29042_MOESM1_ESM.docx]

**Supplementary Appendix**

*To paper Waldauf et al.: Machine learning determination of motivators of terminal extubation during the transition to end-of-life care in intensive care unit.*

**Contents**

[1. Missing data analysis 2](#_Toc113990274)

[2. Chronic pre-existing medical condition 2](#_Toc113990275)

[3. Brainstem reflexes at WLST 3](#_Toc113990276)

[4. Vasoactive drugs 3](#_Toc113990277)

[5. Exploratory data analysis of features by centre 4](#_Toc113990278)

[6. Correlation between features 6](#_Toc113990279)

[7. Logistic regression model 6](#_Toc113990280)

[8. Performance of random forest and logistic regression models 7](#_Toc113990281)

[9. Feature importance of random forest model 7](#_Toc113990282)

[10. Partial dependence plots of random forest model 9](#_Toc113990283)

[11. Overall interaction strength of random forest model 10](#_Toc113990284)

[12. Kaplan-Meier survival curves 11](#_Toc113990285)

[13. Principal component analysis of features 12](#_Toc113990286)

[14. Ethics Approval by Site 12](#_Toc113990287)

[15. Centres 13](#_Toc113990288)

# Missing data analysis


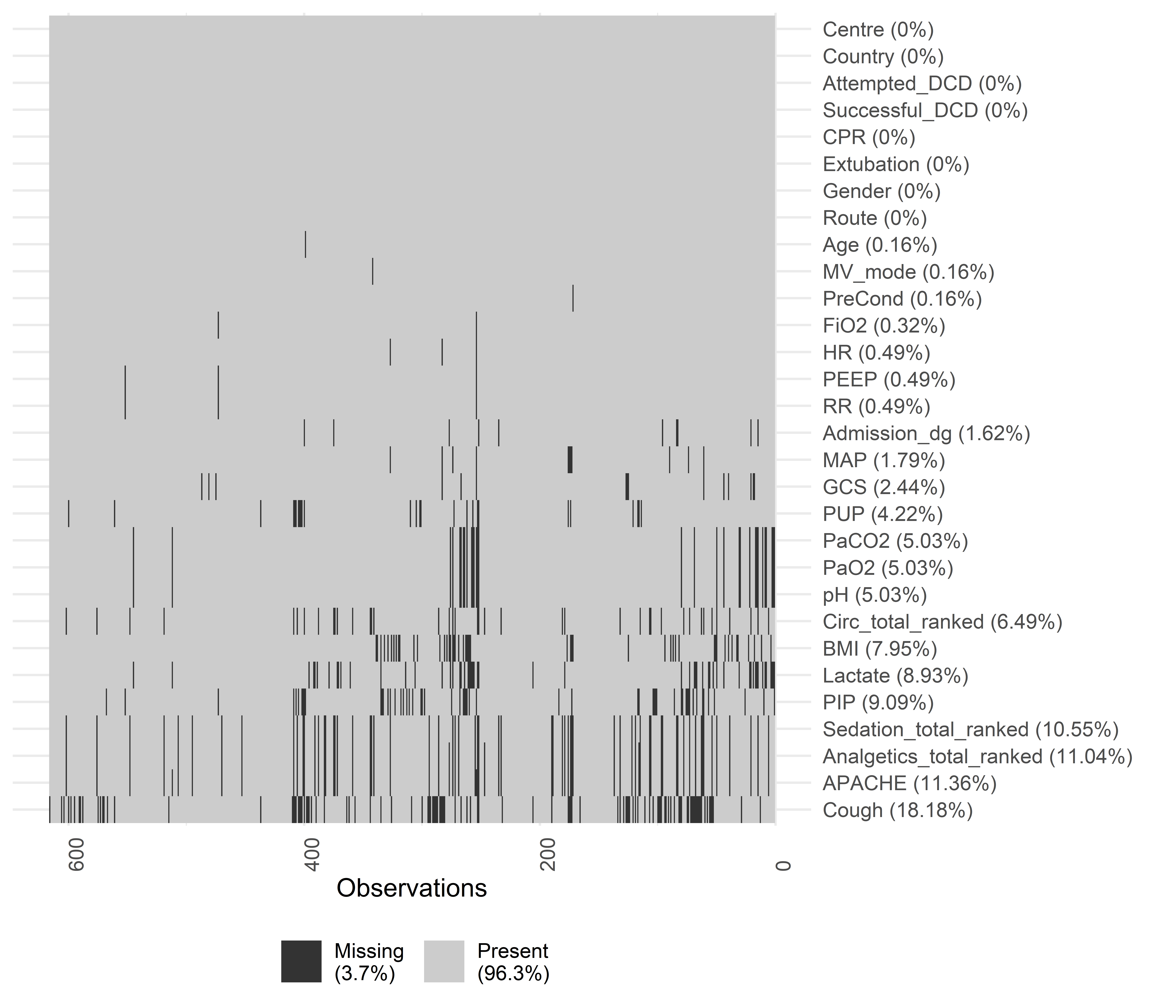


Figure S1: Missing data in the dataset,

black indicates a missing cell, grey indicates a present cell

# Chronic pre-existing medical condition

Table S1: Chronic pre-existing medical condition

| Chronic pre-existing medical condition | Count and percentage |
| --- | --- |
| Cardiovascular disease | 297 (48.3%) |
| Endocrine disease | 157 (25.5%) |
| Chronic Lung disease | 131 (21.3%) |
| GI disease | 98 (15.9%) |
| Cancer / Oncologic disease | 91 (14.8%) |
| Neurologic disease | 76 (12.4%) |
| Renal disease | 62 (10.1%) |
| Psychiatric disorder | 44 (7.2%) |
| Musculo-skeletal disease | 37 (6.0%) |

# Brainstem reflexes at WLST

Table S2: Brainstem reflexes at WLST (^*^ percentages calculated from filled values only)

| Brainstem reflex | Present | Absent | Disagreement between the right and left side | Missing values |
| --- | --- | --- | --- | --- |
| Pupillary r. | 415 (65.9%, 68.8%^*^) | 184 (29.9%, 31.2%^*^) | 24 (4.1%^*^) | 26 (4.2%) |
| Corneal r. | 228 (37.1%, 66.9%^*^) | 113 (18.04%, 33.1%^*^) | 0 | 274 (44.6%) |
| Cough | 360 (58.5%, 71.6%^*^) | 143 (23.3%, 28.4%^*^) | - | 112 (18.2%) |
| Oculocephalic r. | 39 (6.3%, 55,7%^*^) | 31 (5.0%, 44.3%^*^) | 2 (2.7%^*^) | 542 (88.6%) |
| Oculovestibular r. | 17 (2.8%, 47.2%^*^) | 19 (3.1%, 52.8%^*^) | 0 | 579 (94,1%) |

# Vasoactive drugs

Table S3: Vasoactive drugs,

the dose is calculated only for patients with vasoactive,

^*^ percentages calculated from filled values only,

^**^ 18 patients with missing records were noted to have no vasoactive substances

| Drug | Dose  mean ± SD | Dose  median (25;75 percentile) | Number (%) of patients | Missing values |
| --- | --- | --- | --- | --- |
| Norepinephrine [µg min^-1^] | 47.3 ± 65.7 | 23.2 (9.4; 60.1) | 264 (42.9%, 48.3%^*^) | 68 (11.1%) |
| Epinephrine [µg min^-1^] | 21.1 ± 32 | 12 (10; 20) | 23 (3.7%, 4.2%^*^) | 68 (11.1%) |
| Vasopressin [IU min^-1^] | 2.9 ± 2.9 | 2.4 (2.4; 2.4) | 61 (9.9%, 11.2%^*^) | 68 (11.1%) |
| Phenylephrine [µg min^-1^] | 188.4 ± 98.3 | 200 (105; 300) | 26 (4.2%, 4.8%^*^) | 68 (11.1%) |
| Total ranked dose | 0.66 ± 0.52 | 0.62 (0.29; 0.88) | 285 (46.3%, 49.6%^*^) | 50 (6.5%)^**^ |


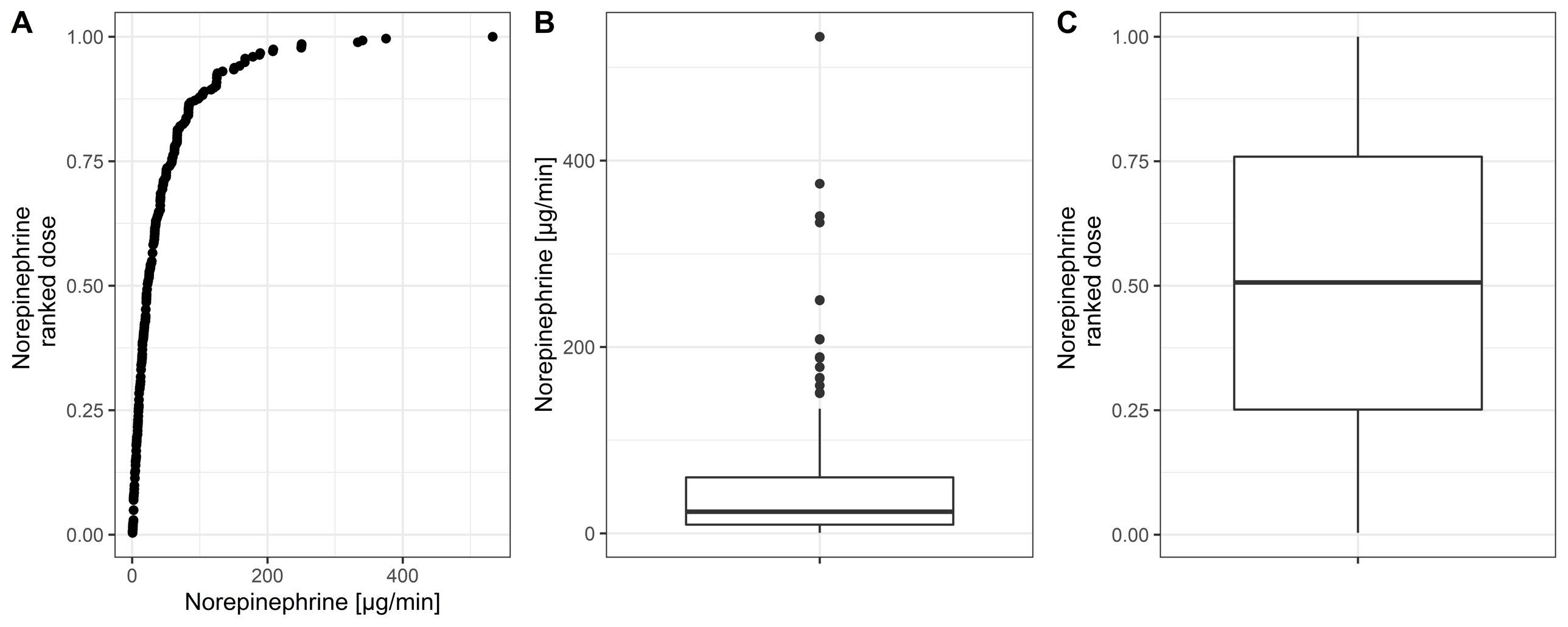


Figure S2: Norepinephrine dose

A: The relationship between norepinephrine dose in µg min^-1^ with ranked dose of norepinephrine

B: Boxplot - distribution of norepinephrine dose in µg min^-1^

C: Boxplot - distribution of ranked dose of norepinephrine

# Exploratory data analysis of features by centre

Table S4: Exploratory data analysis of features by centre (imputed data),

continues features: median (IQR), ^*^ Wilcoxon rank sum test,

categorical features: n (%), ^**^ Pearson’s Chi-squared test

^†^ Parameters that are not part of models

| Feature | All Patients  (N = 616) | Group 1  N = 271 (44%) | Group 2  N = 345 (56%) | p value |
| --- | --- | --- | --- | --- |
| Characteristics of Enrolled Patients at Baseline | | | | |
| Country |  |  |  | <0.001 ^**^ |
| Canada | 355.0 (57.6%) | 52 (19.2%) | 303 (87.8%) |  |
| Czech Republic | 219.0 (35.6%) | 219 (80.8%) | 0 (0.0%) |  |
| Netherlands | 42.0 (6.8%) | 0 (0.0%) | 42 (12.2%) |  |
| Age | 65 (56, 75) | 68 (58, 77) | 63 (54, 73) | <0.001 ^*^ |
| Sex |  |  |  | 0.5 ^**^ |
| Females | 233.0 (37.8%) | 107 (39.5%) | 126 (36.5%) |  |
| Males | 383.0 (62.2%) | 164 (60.5%) | 219 (63.5%) |  |
| Chronic pre-existing medical condition | 507.0 (82.3%) | 235 (86.7%) | 272 (78.8%) | 0.011 ^**^ |
| Cardiac arrest with resuscitation before study inclusion | 84.0 (13.6%) | 56 (20.7%) | 28 (8.1%) | <0.001 ^**^ |
| Admission diagnosis |  |  |  |  |
| Neurologic disorder | 302.0 (49.0%) | 108 (39.9%) | 194 (56.2%) | <0.001 ^**^ |
| Respiratory failure | 94.0 (15.3%) | 43 (15.9%) | 51 (14.5%) | 0.7 ^**^ |
| Sepsis | 91.0 (14.8%) | 58 (21.4%) | 33 (9.6%) | <0.001 ^**^ |
| Other | 129 (20.9%) | 62 (22.9%) | 67 (19.4%) | 0.3 ^**^ |
| Active malignancy ^†^ | 96.0 (15.6%) | 56.0 (20.7%) | 40.0 (11.6%) | 0.002 ^**^ |
| Metastatic malignancy ^†^ | 30.0 (4.9%) | 21.0 (7.7%) | 9.0 (2.6%) | 0.003 ^**^ |
| BMI | 27 (24, 31) | 28 (23, 31) | 27 (24, 30) | >0.9 ^*^ |
| APACHE II score (1st 24 hrs at ICU) | 27 (22, 32) | 29 (22, 34) | 27 (22, 31) | 0.01 ^*^ |
| Characteristics of Enrolled Patients at WLST | | | | |
| Glasgow Coma Scale | 3 (3, 5) | 3 (3, 3.4) | 3. (3, 6) | <0.001 ^*^ |
| Pupillary reflex |  |  |  | 0.039 ^**^ |
| Present | 428.0 (69.5%) | 200 (73.8%) | 228 (66.1%) |  |
| Absent | 188.0 (30.5%) | 71 (26.2%) | 117 (33.9%) |  |
| Cough |  |  |  | 0.001 ^**^ |
| Present | 449.0 (72.9%) | 215 (79.3%) | 234 (67.8%) |  |
| Absent | 167.0 (27.1%) | 56 (20.7%) | 111 (32.2%) |  |
| Ventilation mode |  |  |  | 0.9 ^**^ |
| Support | 175.0 (28.4%) | 76 (28.0%) | 99 (28.7%) |  |
| Control | 441.0 (71.6%) | 195 (72.0%) | 246 (71.3%) |  |
| Respiratory rate [bpm] | 20 (16, 25) | 20 (16, 25) | 20 (16, 26) | 0.5 ^*^ |
| FiO2 [%] | 40 (30, 60) | 40 (33, 60) | 40 (30, 55) | >0.9 ^*^ |
| PEEP [cmH_2_O] | 8 (6, 10) | 8 (6, 10) | 8 (5, 10) | 0.003 ^**^ |
| Peak inspiratory pressure [cmH_2_O] | 22 (18, 27) | 22 (17, 27) | 23 (18, 28) | 0.007 ^*^ |
| Route |  |  |  | 0.087 ^**^ |
| Endotracheal tube | 597.0 (96.9%) | 259 (95.6%) | 338 (98.0%) |  |
| Trache | 19.0 (3.1%) | 12 (4.4%) | 7 (2.0%) |  |
| Mean arterial pressure [mmHg] | 73 (62, 90) | 70 (57, 82) | 77 (66, 95) | <0.001 ^*^ |
| Heart rate [bpm] | 91 (75, 108) | 96 (80, 113) | 87 (73, 105) | <0.001 ^*^ |
| Lactate [mmol L^-1^] | 1.8 (1.1, 3.8) | 2.0 (1.1, 7.0) | 1.7 (1.1, 2.8) | 0.005 ^*^ |
| pH (arterial) | 7.39 (7.28, 7.45) | 7.36 (7.24, 7.44) | 7.41 (7.33, 7.45) | <0.001 ^*^ |
| pO2 [mmHg] (arterial) | 95 (76, 118) | 87 (73, 108) | 100 (81, 131) | <0.001 ^*^ |
| pCO2 [mmHg] (arterial) | 39 (34, 45) | 40 (34, 47) | 38 (34, 45) | 0.079 ^*^ |
| Total ranked dose of circulatory drugs | 0.05 (0.00, 0.61) | 0.39 (0.0, 0.78) | 0.0 (0.0, 0.29) | <0.001 ^*^ |
| Patients with circulatory drugs ^†^ | 325.0 (52.8%) | 181.0 (66.8%) | 144.0 (41.7%) | <0.001 ^**^ |
| Total ranked dose of circulatory drugs (only patients with circulatory drugs) † | 0.59 (0.26, 0.85) | 0.66 (0.39, 0.89) | 0.41 (0.18, 0.78) | <0.001 ^*^ |
| Total ranked dose of sedatives | 0.00 (0.00, 0.37) | 0.05 (0.0, 0.24) | 0.0 (0.0, 0.49) | <0.001 ^*^ |
| Patients with sedatives ^†^ | 270.0 (43.8%) | 99.0 (36.5%) | 171.0 (49.6%) | 0.001 ^**^ |
| Total ranked dose of sedatives (only patients with sedatives) ^†^ | 0.47 (0.22, 0.81) | 0.40 (0.21, 0.76) | 0.50 (0.22, 0.81) | 0.2 ^*^ |
| Total ranked dose of opioids | 0.04 (0.00, 0.49) | 0.0 (0.0, 0.33) | 0.20 (0.0, 0.56) | <0.001 ^*^ |
| Patients with opioids † | 313.0 (50.8%) | 114.0 (42.1%) | 199.0 (57.7%) | <0.001 ^**^ |
| Total ranked dose of opioids (only patients with opioids) ^†^ | 0.48 (0.26, 0.80) | 0.47 (0.21, 0.73) | 0.49 (0.30, 0.84) | 0.055 ^*^ |
| Eligible for Donation after Circulatory Death † | 307.0 (49.8%) | 96.0 (35.4%) | 211.0 (61.2%) | <0.001 ^**^ |
| Attempted Donation after Circulatory Death | 87.0 (14.1%) | 7 (2.6%) | 80 (23.2%) | <0.001 ^**^ |
| Successful Donation after Circulatory Death | 60.0 (9.7%) | 6 (2.2%) | 54 (15.7%) | <0.001 ^**^ |
| Extubation | 396.0 (64.3%) | 84 (31.0%) | 312 (90.4%) | <0.001 ^**^ |

# Correlation between features


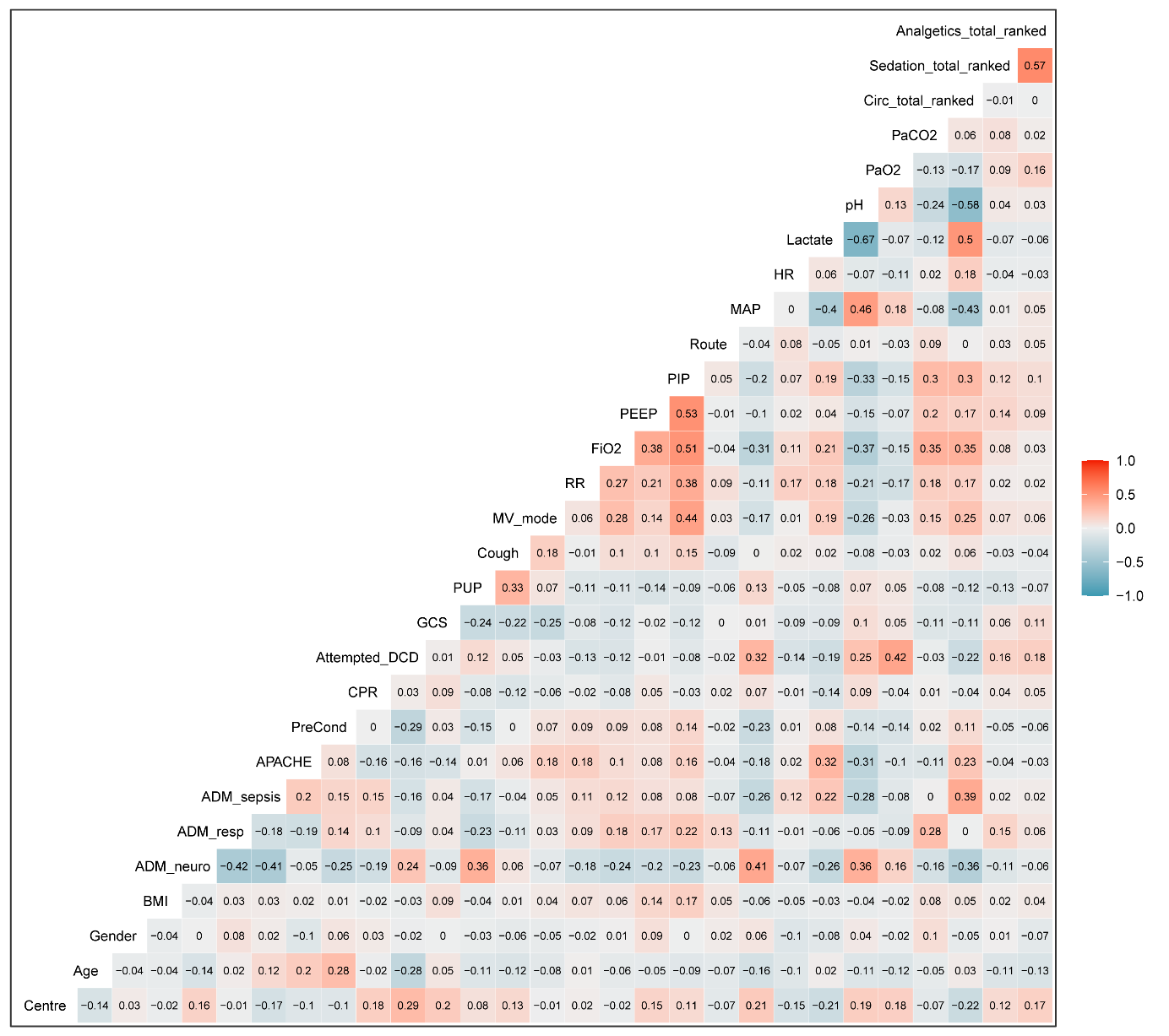


Figure S3: Pearson correlation between features used in the RF and LR models

None of the correlations reached the empirically chosen cut point R > 0.7 or <-0.7 so that the feature had to be excluded from the modelling due to multicollinearity.

# Logistic regression model

Table S5: Logistic regression model with feature selection (Lasso regularisation)

| Feature | Adjusted odds ratio  (95% confidence interval) | p value |
| --- | --- | --- |
| Centre (group 2) | 34.9 (19.7; 65.4) | <0.001 |
| Ranked total circulatory drugs dose at WLST (cont.) | 0.49 (0.26; 0.88) | 0.019 |
| Peak inspiratory pressure [cmH_2_O] at WLST (cont.) | 0.94 (0.90; 0.98) | 0.005 |
| Lactate [mmol L^-1^] at WLST (cont.) | 0.93 (0.87; 0.98) | 0.006 |
| Mean arterial pressure [mmHg] at WLST (cont.) | 1.02 (1.003; 1.031) | 0.021 |
| APACHE II at admission (cont.) | 1.04 (1.004; 1.07) | 0.029 |
| Route (trache vs orotracheal intubation) | 0.28 (0.07; 0.95) | 0.044 |
| Admission diagnosis: neurological disorder vs other | 1.54 (0.90; 2.63) | 0.112 |
| FiO_2_ at WLST (cont.) | 0.99 (0.98; 1.003) | 0.129 |

# Performance of random forest and logistic regression models

Table S6: Performance in 5-fold cross-validation repeated 5-times

|  | Featureless model  (no feature) | Random forest  (all features) | Logistic regression  (all feature) | Logistic regression (Lasso) |
| --- | --- | --- | --- | --- |
| Classification error | 0.357 | 0.166 | 0.156 | 0.164 |
| ROC AUC | 0.5 | 0.909 | 0.898 | 0.90 |
| Brier score | 0.231 | 0.121 | 0.117 | 0.125 |





Figure S4: Average ROC curve of 5-fold cross-validation repeated 5-times for random forest and logistic regression model with Lasso regularisation

# Feature importance of random forest model

Table S7: Random forest model: permutation feature importance (loss function = classification error (CE)), as the factor by which the model’s CE increases when the feature is shuffled in the test data

|  | Feature | Importance  (permutation CE / original CE (4.7%)) | Permutation CE  (on all data) |
| --- | --- | --- | --- |
| 1 | Centre | 4.517 | 21.3% |
| 2 | Total ranked dose of circulatory drugs at WLST | 1.655 | 7.8% |
| 3 | GCS at WLST | 1.379 | 6.5% |
| 4 | Lactate at WLST [mmol L^-1^] | 1.310 | 6.2% |
| 5 | pH at WLST | 1.310 | 6.2% |
| 6 | Peak inspiratory pressure at WLST [cmH_2_O] | 1.276 | 6.0% |
| 7 | APACHE II score | 1.241 | 5.8% |
| 8 | Age | 1.241 | 5.8% |
| 9 | BMI | 1.241 | 5.8% |
| 10 | Heart rate at WLST [bpm] | 1.241 | 5.8% |
| 11 | Mean arterial pressure at WLST [mmHg] | 1.241 | 5.8% |
| 12 | Respiratory rate at WLST [bpm] | 1.241 | 5.8% |
| 13 | Admission diagnosis: neurologic disorder | 1.207 | 5.7% |
| 14 | PaCO_2_ at WLST [mmHg] | 1.207 | 5.7% |
| 15 | PaO_2_ at WLST [mmHg] | 1.207 | 5.7% |
| 16 | Total ranked dose of opioids at WLST | 1.138 | 5.4% |
| 17 | FiO_2_ at WLST [%] | 1.138 | 5.4% |
| 18 | Admission diagnosis: respiratory failure | 1.103 | 5.2% |
| 19 | PEEP at WLST [mmHg] | 1.103 | 5.2% |
| 20 | Total ranked dose of sedatives at WLST | 1.069 | 5.0% |
| 21 | Cough at WLST | 1.034 | 4.9% |
| 22 | Gender | 1.034 | 4.9% |
| 23 | Pupillary reflex at WLST | 1.034 | 4.9% |
| 24 | Route at WLST | 1.034 | 4.9% |
| 25 | Admission diagnosis: sepsis | 1.000 | 4.7% |
| 23 | Cardiac arrest with resuscitation before study inclusion | 1.000 | 4.7% |
| 27 | Mechanical ventilation mode at WLST | 1.000 | 4.7% |
| 28 | Chronic pre-existing medical condition | 1.000 | 4.7% |
| 29 | Attempted Donation after Circulatory Death | 0.966 | 4.5% |

# Partial dependence plots of random forest model


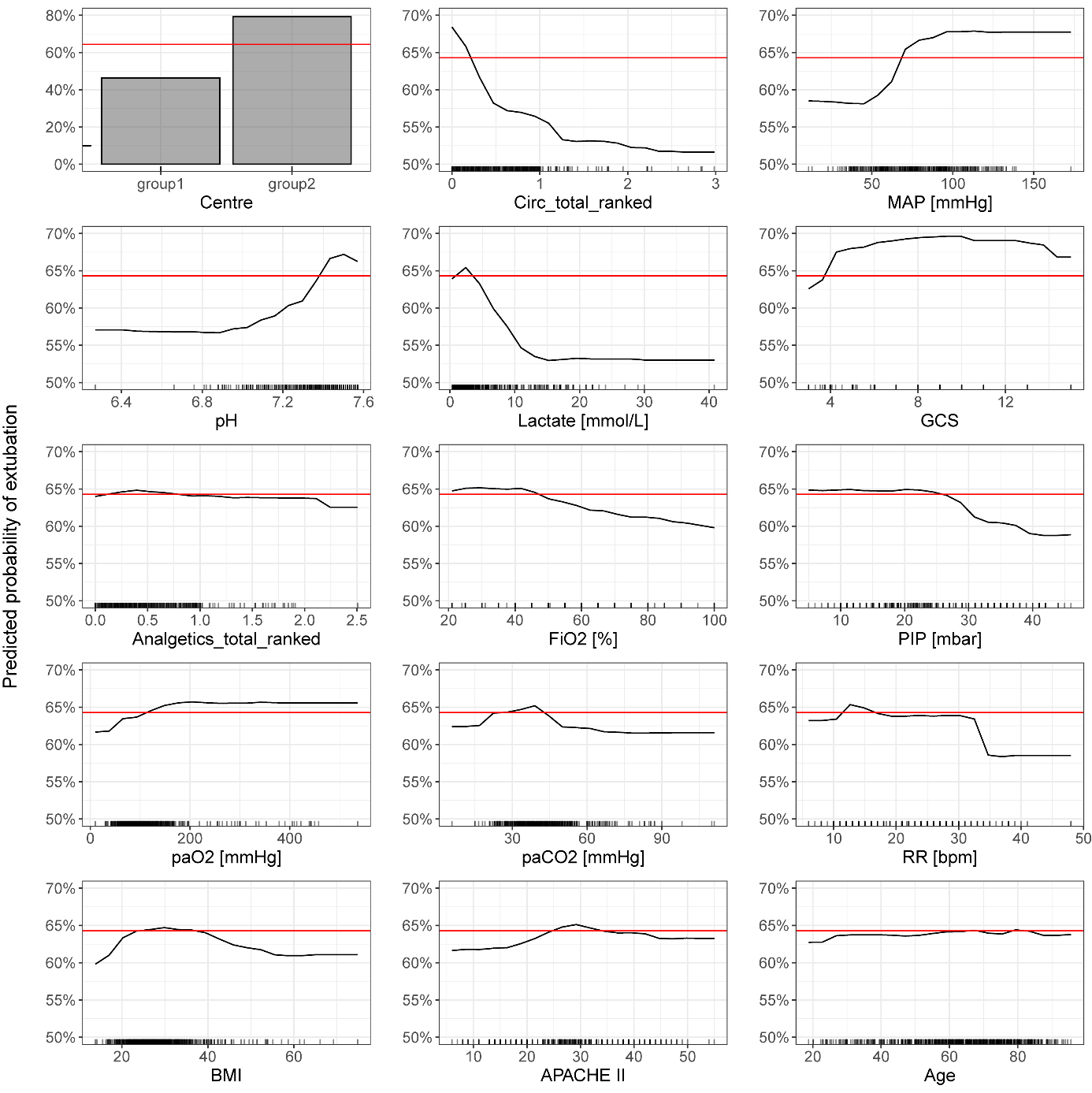


Figure S5: Random forest model (15 most important features): partial dependence plots (PDP) showing marginal effects of features, that means how the probability of terminal extubation changes as the value of a feature changes while considering the average effect of all the other features in the model, red horizontal line is average probability of terminal extubation (64.3%)

# Overall interaction strength of random forest model


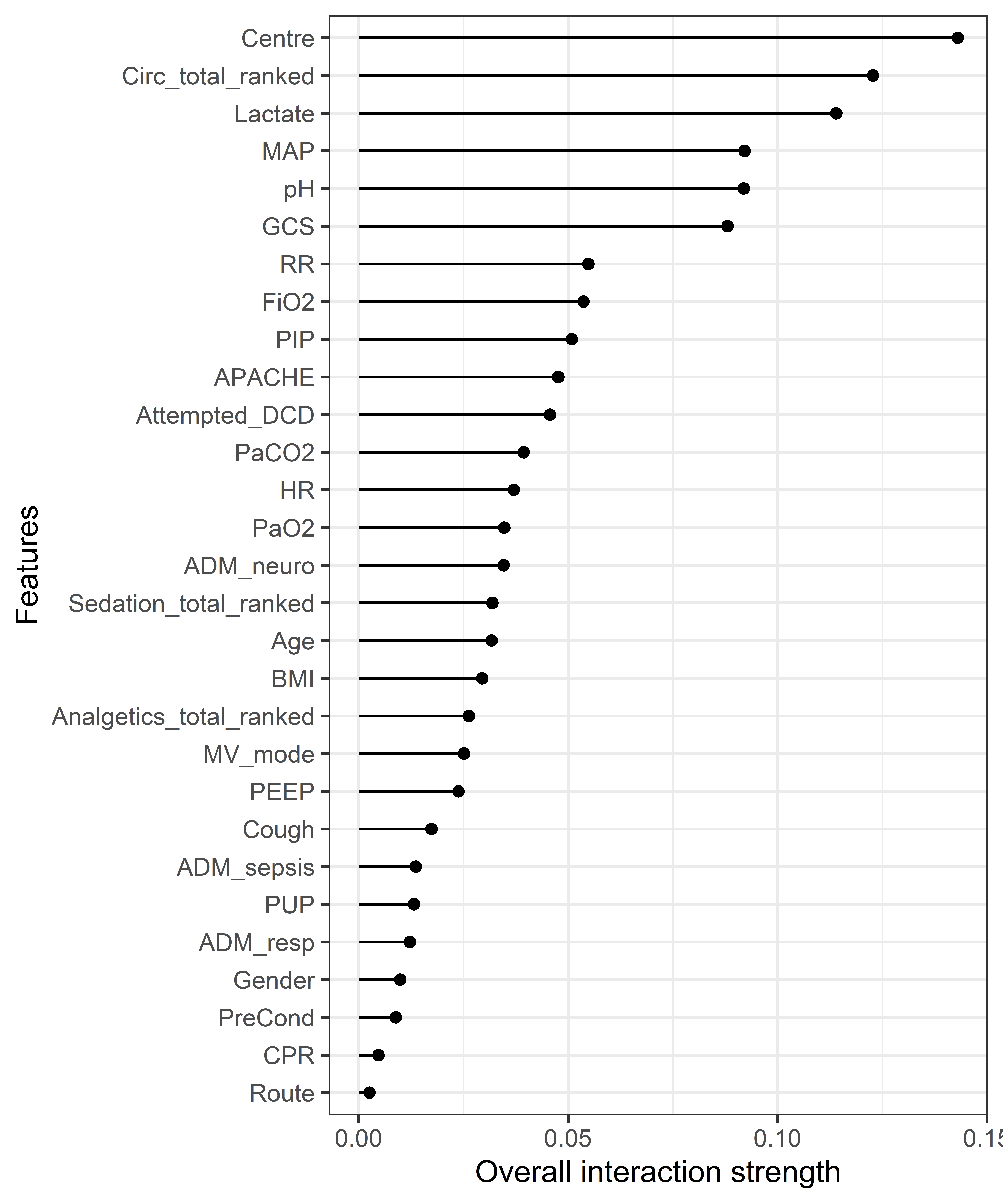


Figure S6: Random forest model: overall interaction strength for each feature with all other features overall, the interaction effects between the features are very weak (below 10% of variance explained per feature)


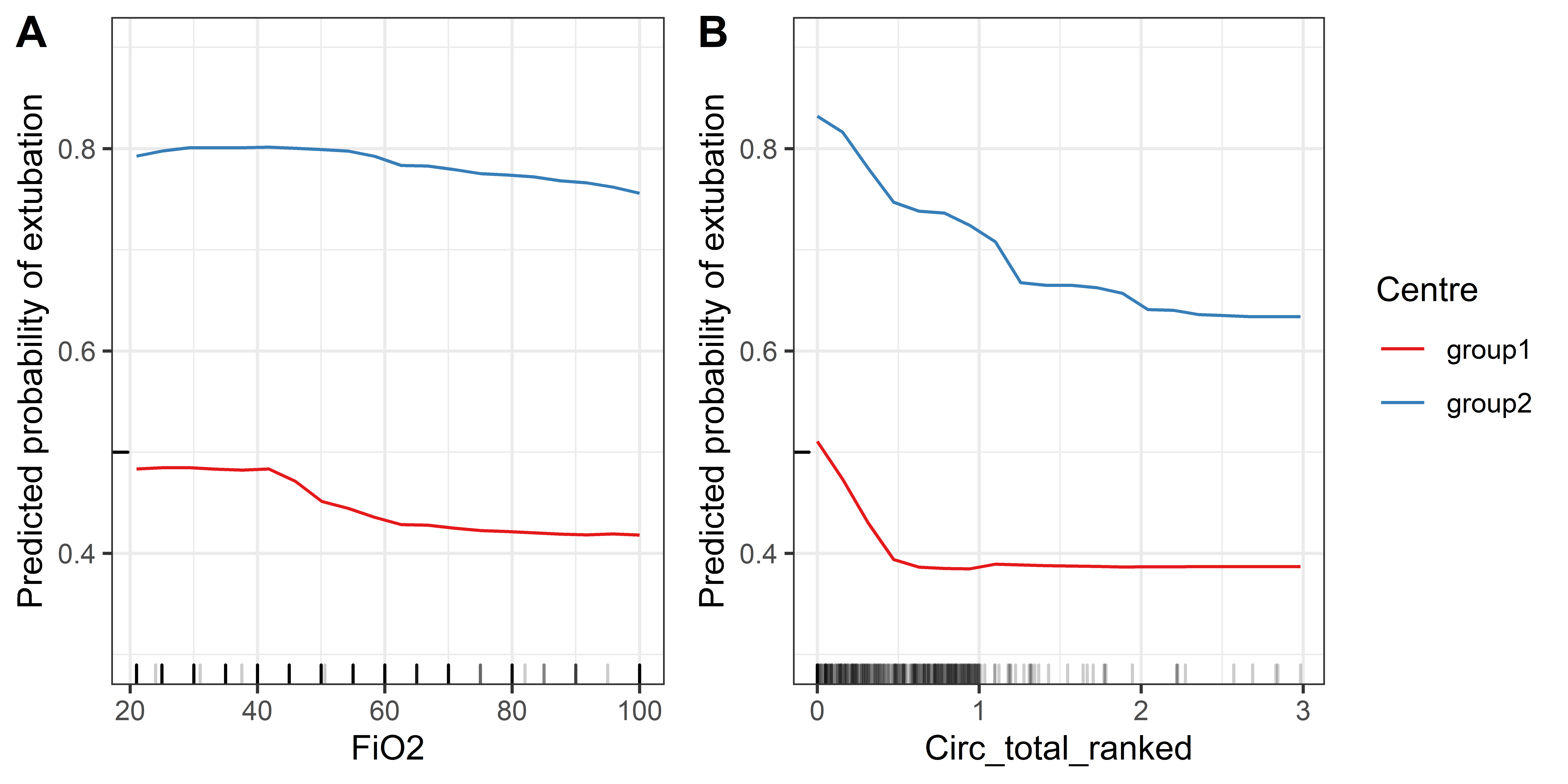


Figure S7: Random forest model, partial dependence plots (PDP): interaction between centre group and two features (A: FiO2 and B: Circ_total_ranked) (the effect of FiO_2_ and total dose of ranked circulating drugs dose on probability of terminal extubation in group 1 and 2)

# Kaplan-Meier survival curves


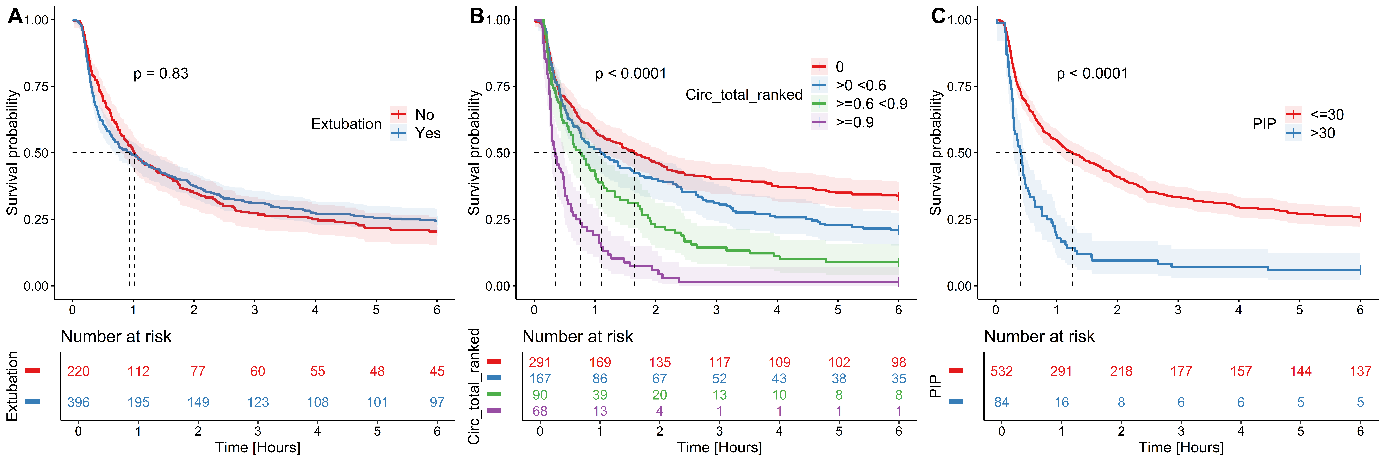


Figure S8: Kaplan-Meier survival curve, p-value comes from log-rank test

A: Patients with and without terminal extubation

B: Patients with different total circulatory drugs doses

C: patients with peak inspiratory pressure above and below 30 cmH_2_O

# Principal component analysis of features





Figure S9: Principal component analysis (PCA) based on the first two principal components (Dim1 and Dim2) of all features

A: The correlation between each feature and a principal component 1 and 2 (positively correlated variables are grouped together, negatively correlated variables are positioned on opposite sides of the plot origin, the distance between variables and the origin measures the quality of the variables on the map)

B: The same graph shows individual patients colour coded according to terminal extubation (blue triangle = terminal extubation, red dot = without terminal extubation)

Note: PCA is a mathematical technique frequently used to reduce the number of features used in machine learning algorithms. As can be seen from the correlation plot (Figure S3) many features are mutually associated. PCA produces a smaller number of features/dimensions with zero association from all features. Here, PCA is chosen to visualize univariate relationships between features and terminal extubation. The plots show the 1st 2 most important dimensions (principal components) explaining 16% (x-axis) and 9% (y-axis) of the variability in the feature dataset, respectively. Graph B shows how the 1st and 2nd dimensions segment patients by extubation reasonably well. Those who were extubated are mainly in the left part of the graph (blue triangles). Graph A shows the individual features and how they are distributed relative to the 1st and 2nd dimensions. Comparing graphs, A and B, can be seen how the individual features apply to extubation - depending on where the arrows point - whether they are more to the side of extubation or against it.

# Ethics Approval by Site

Table S8: Ethics Approval by Site

| CHEO Research Ethics Board | No. 14/08E |
| --- | --- |
| Ottawa Health Science Network Research Ethics Board | Protocol # 20140337-01H |
| St. Michael’s Hospital Research Ethics Board | REB# 14-335 |
| Western University Health Science Research Ethics Board | HSREB File Number 105752 |
| University of Alberta Health Research Ethics Board | Study ID Pro00063243 |
| Nova Scotia Health Authority Research Ethics Board | NSHA REB ROMEO File # 1020827 |
| Sunnybrook Health Sciences Centre Research Ethics Board | Protocol ID # 042-2015 |
| University of British Columbia Clinical Research Ethics Board | UBC CREB # H14-02114 |
| Hamilton Integrated Research Ethics Board | REB Project # 14-405 |
| McGill University Health Centre Genetics/Population Research Ethics Board | #14-204 GEN |
| University of Calgary Conjoint Health Research Ethics Board | Ethics ID REB14-1337 |
| Mount Sinai Hospital Research Ethics Board | #14-0340-E |
| Queen’s University Health Sciences & Affiliated Teaching Hospitals Research Ethics Board (HSREB) | ROMEO/TRAQ# 6018723 |
| Ethics Committee of the General University Hospital, Prague | #503/15 S-IV |
| Ethics Committee of the Medical Faculty of Charles University | EK-VP/68/0/2014 |
| Maastricht UMC REB | METC 16-4-174 |

# Centres

Table S9: List of centres included in the study

| Name | Location | Type of ICU | Centre # |
| --- | --- | --- | --- |
| Calgary Foothills – Rockyview General Hospital | Calgary, AB, Canada | General medical/surgical | 18 |
| Calgary Foothills – South Health Campus | Calgary, AB, Canada | General medical/surgical | 17 |
| Calgary Foothills Hospital | Calgary, AB, Canada | General medical/surgical | 12 |
| University of Alberta Hospital | Edmonton, AB, Canada | General medical/surgical | 5 |
| Queen Elizabeth II Hospital | Halifax, NS, Canada | General medical/surgical | 6 |
| Hamilton General Hospital | Hamilton, ON, Canada | General medical/surgical | 10 |
| Kingston General Hospital | Kingston, ON, Canada | General medical/surgical | 16 |
| London Health Sciences Centre – University | London, ON, Canada | Neuro-ICU | 4 |
| London Health Sciences Centre – Victoria | London, ON, Canada | General medical/surgical | 15 |
| Montreal General Hospital | Montreal, QC, Canada | General medical/surgical | 11 |
| The Ottawa Hospital- Civic Campus | Ottawa, ON, Canada | Trauma | 14 |
| The Ottawa Hospital- General Campus | Ottawa, ON, Canada | General medical/surgical | 2 |
| Mount Sinai Hospital | Toronto, ON, Canada | General medical/surgical | 13 |
| St. Michael’s Hospital | Toronto, ON, Canada | General medical/surgical Trauma | 3 |
| Sunnybrook Hospital | Toronto, ON, Canada | Trauma | 7 |
| Vancouver General Hospital | Vancouver, BC, Canada | General medical/surgical | 9 |
| Maastricht University Medical Centre | Maastricht, Netherlands | General medical/surgical | 61 |
| Fakultni Nemonice Kralovske Vinohrady – ICU II | Prague, Czech Republic | General medical/surgical | 83 |
| Fakultni Nemonice Kralovske Vinohrady – ICU I | Prague, Czech Republic | General medical/surgical/trauma | 82 |
| Vseobecna fakultni nemocnice | Prague, Czech Republic | General medical/surgical | 81 |
